# Strategic testing approaches for targeted disease monitoring can be used to inform pandemic decision-making

**DOI:** 10.1371/journal.pbio.3001307

**Published:** 2021-06-17

**Authors:** James D. Nichols, Tiffany L. Bogich, Emily Howerton, Ottar N. Bjørnstad, Rebecca K. Borchering, Matthew Ferrari, Murali Haran, Christopher Jewell, Kim M. Pepin, William J. M. Probert, Juliet R. C. Pulliam, Michael C. Runge, Michael Tildesley, Cécile Viboud, Katriona Shea

**Affiliations:** 1 U.S. Geological Survey, Eastern Ecological Science Center at the Patuxent Research Refuge, Laurel, Maryland, United States of America; 2 Department of Biology, The Pennsylvania State University, University Park, Pennsylvania, United States of America; 3 The Center for Infectious Disease Dynamics, The Pennsylvania State University, University Park, Pennsylvania, United States of America; 4 Department of Entomology, The Pennsylvania State University, University Park, Pennsylvania, United States of America; 5 Department of Statistics, The Pennsylvania State University, University Park, Pennsylvania, United States of America; 6 Lancaster Medical School, Lancaster University, Lancaster, United Kingdom; 7 National Wildlife Research Center, United States Department of Agriculture, Animal and Plant Health Inspection Service, Wildlife Services, Fort Collins, Colorado, United States of America; 8 Big Data Institute, Nuffield Department of Medicine, University of Oxford, Oxford, United Kingdom; 9 South African DSI-NRF Centre of Excellence in Epidemiological Modelling and Analysis (SACEMA), Stellenbosch University, Cape Town, Western Cape, South Africa; 10 Zeeman Institute: Systems Biology and Infectious Disease Epidemiology Research (SBIDER), Mathematics Institute and School of Life Sciences, University of Warwick, Coventry, United Kingdom; 11 Fogarty International Center, National Institutes of Health, Bethesda, Maryland, United States of America

## Abstract

More than 1.6 million Severe Acute Respiratory Syndrome Coronavirus 2 (SARS-CoV-2) tests were administered daily in the United States at the peak of the epidemic, with a significant focus on individual treatment. Here, we show that objective-driven, strategic sampling designs and analyses can maximize information gain at the population level, which is necessary to increase situational awareness and predict, prepare for, and respond to a pandemic, while also continuing to inform individual treatment. By focusing on specific objectives such as individual treatment or disease prediction and control (e.g., via the collection of population-level statistics to inform lockdown measures or vaccine rollout) and drawing from the literature on capture–recapture methods to deal with nonrandom sampling and testing errors, we illustrate how public health objectives can be achieved even with limited test availability when testing programs are designed a priori to meet those objectives.

## Abstract 2

COVID-19 testing programs are very important to help control the pandemic. In this Essay, the authors show that objective-driven, strategic sampling designs and analytics can be used to maximize the information gained by COVID-19 testing programs and improve population-level decisions, while maintaining the value of these programs for patient-level management.

## Introduction

“Did you lose the keys here? No, but the light is much better here.” (Streetlight metaphor, various attributions)

As we await widespread access to vaccines globally and manage delays in vaccine rollout (e.g., [1,2]), testing—used in conjunction with contact tracing and isolation—is a critical tool for controlling the spread of Severe Acute Respiratory Syndrome Coronavirus 2 (SARS-CoV-2) [3], understanding the dynamics of more contagious variants [4], and planning for future outbreaks [5]. While testing for the virus is key to limiting transmission by enabling the early detection and control of local outbreaks and informing vaccination strategies by providing the parameter estimates needed for epidemiological modeling (“population-level” objectives), tests are still primarily used for individual treatment (“individual-level” objectives). Internationally, there are some examples of testing efforts to inform population-level objectives (e.g., [6]). Despite widespread agreement on the need for more, and more coordinated, testing [7,8], such efforts at the national scale appear to be lacking in the US, especially testing to inform population-level objectives critical to pandemic vaccine rollout. With a limited, albeit growing, number of tests, we must carefully consider who, when, where, and how often to test for virus presence and how to interpret results to inform differing public health objectives (Table 1) [9,10]. In this paper, we argue that current testing approaches could be further strengthened with the strategic allocation of relatively few additional tests and symptom-based surveys. We also argue that this approach is critical to the development of targeted disease monitoring for national programs such as the proposed National Center for Epidemic Forecasting and Outbreak Analytics [5]. We focus on testing within the US public health system in particular but expect the proposed approach to apply more broadly.

**Table 1:** Examples of objective-driven sampling strategies and their utility for individual-level versus population-level inferences.

Objective	Test utility	Sampling design
*Individual level*		
Therapeutic	Determine infection status and appropriate medical treatment for a symptomatic individual	Test symptomatic individuals who self-report or individuals in high-risk categories
Contact tracing	Trigger the process of identifying persons with whom a known infected individual has been in recent contact to test and/or quarantine contacts who may have been infected and limit spread	Test (typically) symptomatic individuals, with subsequent tests allocated to individuals with whom the focal individuals have had contact
Prophylactic	Determine infection status to inform entry permission (e.g., to a workplace, airline flight, school, or event space) and decrease risk of transmission to others in the specified group or location; determine precautions for healthcare professionals (e.g., PPE)	Test all individuals associated with the focal location or group and repeat periodically (e.g., for workplaces, schools, or recurring events)
*Population level*		
Epidemiological	Estimate key epidemiological parameters (e.g., prevalence, mortality rate^a^, and infection rate^a^) to investigate disease dynamics and parameterize projection models	Select a random or representative subset from the population to test (or nonrepresentative subsets and estimate sampling probabilities)
Decision-making	Determine effective vaccine distribution within and between populations, assess risk for hospital planning and resource allocation (e.g., beds, ventilators, and PPE), or evaluate the effectiveness of a public health policy aimed at reducing transmission (e.g., mask wearing, distancing, nonessential business closures, etc.) based on context-dependent epidemiological parameters (e.g., prevalence, mortality rate^a^, and infection rate^a^)	Select a random or representative subset from the population to test (or nonrepresentative subsets and estimate sampling probabilities)

^a^Inference requires follow-up testing of sampled individuals.

PPE, personal protective equipment.

### Objective-driven sampling

**Fig 1:**
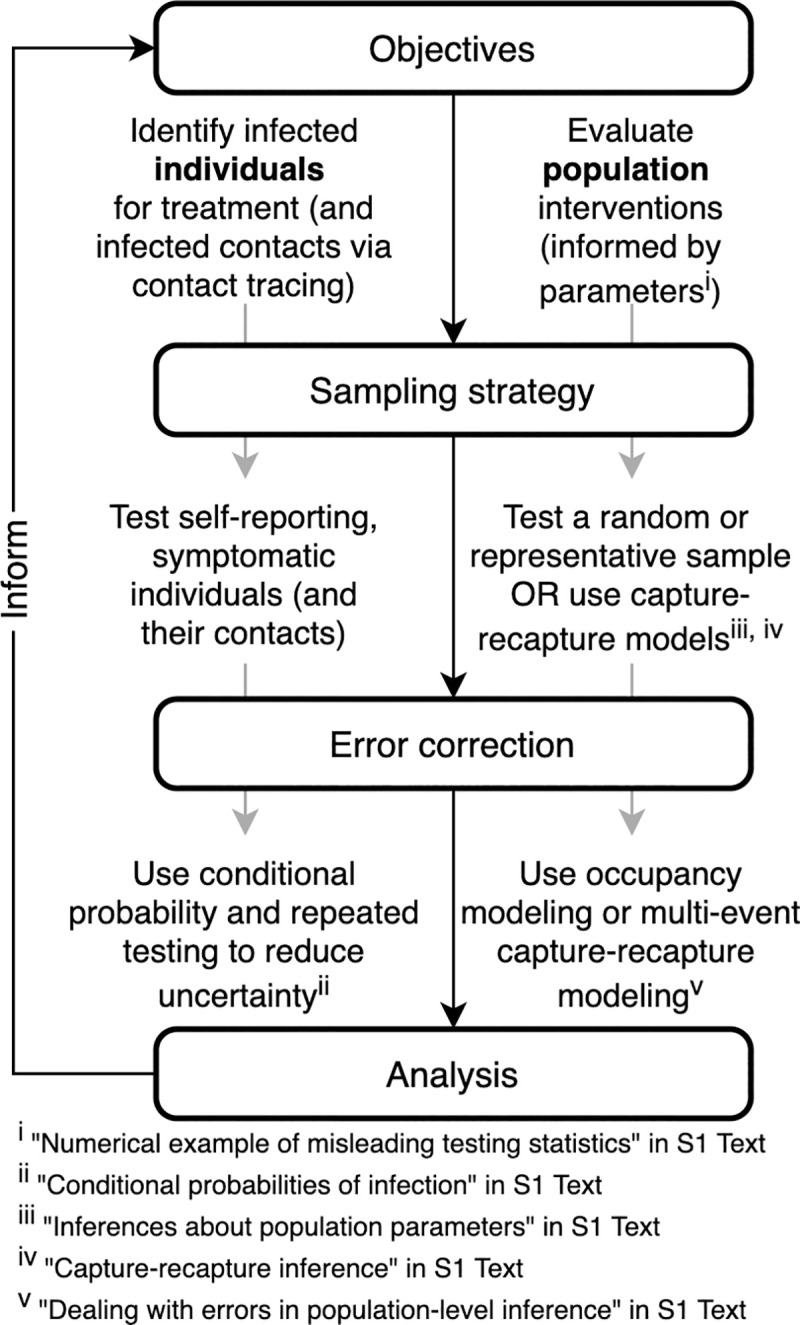
Objective-driven testing framework. Testing strategy design, “sampling strategy,” is part of a multistep framework, including error correction and analysis to inform individual- or population-level public health objectives.

We use the “streetlight effect” metaphor (of searching where convenient) to suggest potential problems with our use of collected statistics on Coronavirus Disease 2019 (COVID-19) cases for all of the various decisions requiring data. These kinds of problems extend beyond the current pandemic to a variety of disciplines and issues for which omnibus monitoring programs are used to meet all potential monitoring needs. We advocate an alternative approach that focuses sample design and parameter estimation (including error correction) on meeting specific objectives (see Fig 1). The proposed approach does not preclude the use of such targeted data for secondary objectives, when appropriate, but instead seeks to ensure that at least the primary objective(s) can be met. Further, this approach does not necessarily require the collection of more data, but the targeted, more efficient collection of data for specified objectives. The importance of tailoring sampling strategies to the question or parameter of interest has been demonstrated previously (e.g., for ecological monitoring as in [11–13], for human disease prevalence as in [14], and for optimal livestock disease control as in [15]). Given that testing for SARS-CoV-2 lacks clear guidelines, we believe there is a need for increased focus on designing test allocation strategies based on the individual-level and population-level objective(s) they are meant to inform (Table 1).

Location-specific numbers of COVID-19 cases and deaths, and inferred quantities such as test positivity rates and death rates, are reported daily. Such reports dominate websites and newsfeeds and are often interpreted as providing comparable information about the pandemic’s trajectory across locations. However, the interpretation and utility of these numbers depend on how individuals are selected for testing and on test result accuracy [9]. For example, comparison of positivity rates or numbers of confirmed cases at 2 locations that use different testing strategies (e.g., testing symptomatic individuals only versus symptomatic and asymptomatic individuals) would likely yield differences that reflect a complicated confounding of true differences in COVID-19 prevalence and artifactual differences due to testing strategy (“Numerical example of misleading testing statistics” in S1 Text) and classification errors (“Conditional probabilities of infection” and “Dealing with errors in population-level inference” in S1 Text). To meet a public health objective requiring such comparisons, we need inference methods that properly account for differences in testing strategy and classification errors.

We propose a strategic framework for thinking about testing in which (1) different objectives of testing are clearly articulated; and (2) sampling design and subsequent data analysis are tailored a priori to achieve these objectives, while accounting for sampling constraints and measurement errors (Fig 1). Our focus is on the use of strategic testing for targeted disease monitoring, in which sampling is designed to provide information used to make treatment or control decisions. We contrast this approach with ad hoc testing, which provides a form of convenience sampling. We illustrate how statistical methods developed primarily in wildlife ecology can be applied to sample design and parameter estimation to meet specified objectives for the current pandemic.

We first discuss a few representative testing objectives relevant to the monitoring of COVID-19 in the pandemic phase. We categorize these objectives as individual- and population-level inferences based on the decisions that test results are intended to inform (Table 1, Fig 1). We focus on the relationship between stated objectives, how individuals are selected for testing (“sampling”), and how errors are handled.

### Inferences about individual parameters

Individual-level inferences entail efforts to assess whether a specific individual is infected with a pathogen (Table 1). Such inferences inform decisions made about the tested individual (e.g., treatment, isolation, etc.). A key concern for these inferences is acting on incorrect results given imperfect diagnostic tests. To account for imperfect tests, decisions can be based on not just the test result (infected or not), but also on conditional probabilities of the result being true or false. Conditional probabilities of test result accuracy (positive or negative predictive values of a test) depend not only on test sensitivity (true positive rate) and specificity (true negative rate), but also on the population-level parameter, prevalence [16,17] (Fig 1 in [18]; “Conditional probabilities of infection” in S1 Text), estimates of which depend on sampling strategy and inference method. If readily measured individual covariates such as symptoms are associated with the probability that an individual is infected, then we model that infection probability as a function of the covariates (see next section) and use it in place of an overall prevalence parameter in the expressions of “Conditional probabilities of infection” in S1 Text.

Conditional probabilities of infection, given either a positive or negative test result, are useful when considering the reasons for seeking individual-level inferences: treatment of the focal individual, quarantine and isolation decisions, safety of attending healthcare workers, or identifying prior contacts of the focal individual. If the computed probabilities admit more uncertainty than desired, then error probabilities often can be reduced with additional information provided by replicating tests on the individual (“Conditional probabilities of infection” in S1 Text).

### Inferences about population parameters

The focus of population-level inference is not on individual test results, but rather on how test results can inform parameter estimates that characterize the entire population (e.g., prevalence, infection rate by age or other characteristics, reproduction number, or disease-specific mortality rate). These estimates are needed to inform epidemiological models and evaluate population-level decisions (e.g., to determine vaccine distribution strategies). Sampling entails the selection of subsets of individuals for testing, and different sampling designs are required for inferences about different population-level parameters such as prevalence (“Inferences about population parameters” in S1 Text).

Prevalence is often defined as the proportion of individuals in a population infected at a given point in time. The only COVID-19 surveillance data available in many countries at present are based on sampling of symptomatic individuals. However, inferences about prevalence and other population-level parameters are not readily extracted from such data [19,20].

When testing resources are limited, prevalence is best estimated by selecting a random or representative (defined with respect to factors influencing the likelihood that an individual is infected) sample of individuals for testing. The fraction of individuals testing positive provides an estimate of prevalence (see “Inferences about population parameters” in S1 Text). But sampling individuals in a random or representative manner is not typical of many standard surveillance programs, and, sometimes, may not be possible at large scales. For example, much of the current information about numbers of COVID-19 cases comes from sampling programs in which symptomatic individuals are tested with much higher probability than asymptomatic individuals.

**Fig 2:**
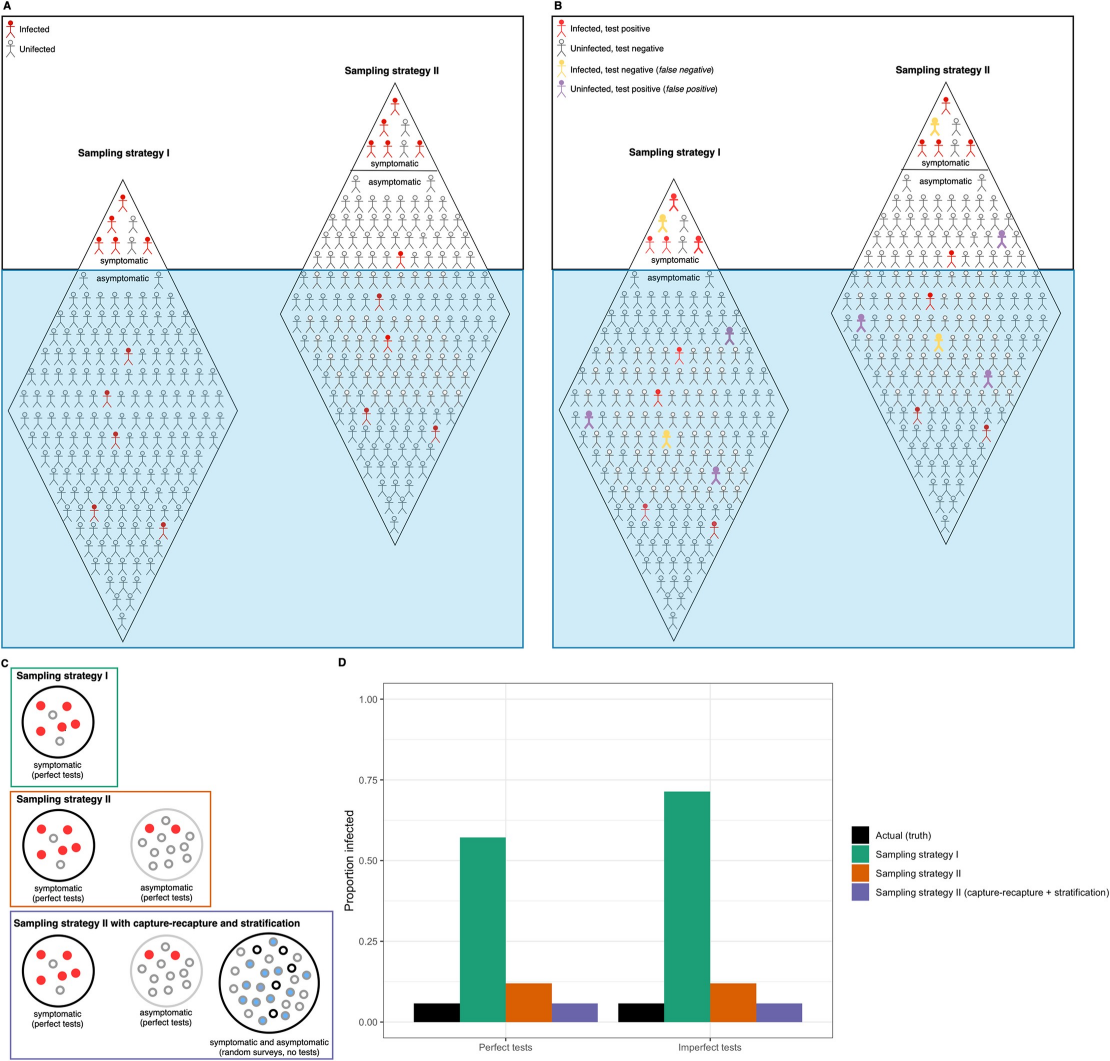
The importance of objective-driven sampling strategy design. The “iceberg” problem is illustrated for 2 different sampling strategies: testing for an objective of inference about whether or not an individual is infected to inform treatment or initiate contact tracing, etc., (sampling strategy I) and testing for an objective of inference about population parameters such as prevalence to inform decision-making about a population-level intervention (sampling strategy II). In both strategies, individuals above the blue “water” line are tested, and those below go untested. We attempt to estimate the prevalence or proportion of individuals infected as the proportion infected for our sample. The total number of infected individuals in both icebergs is the same; however, the proportion infected differs substantially between samples based on the 2 strategies. We illustrate 2 assumptions about test accuracy with the following 4 figure panels: **(A)** Sampling given perfect tests (i.e., the probability of a true positive, *p*_11_, is 1, and the probability of a false positive, *p*_10_, is 0) and **(B)** sampling given imperfect tests. **(C)** We illustrate a third sampling strategy (strategy II with capture–recapture and stratified sampling) and compare it to sampling strategy I (symptomatic individuals only) and II (symptomatic individuals + random sample of asymptomatic individuals). Capture–recapture methods permit approximately unbiased inference in the face of false-negative and positive errors and are combined with stratified sampling to deal with nonrandom sampling. Finally, in **(D)**, we compare the observed proportion infected in the samples based on all 3 strategies to the actual infected proportion of the population (under both scenarios of perfect testing (as in **A**) and imperfect testing (as in **B**)). The application of capture–recapture methods and stratification to strategy II (purple bars) provides the most accurate estimate of the true population prevalence (black bars).

An alternative approach is to select small groups of individuals in a nonrepresentative way and to estimate the probabilities that a randomly sampled individual would appear in these groups (e.g., using symptom-based surveys). These sampling probabilities can be incorporated directly into inference models, permitting approximately unbiased inference despite nonrepresentative sampling (Fig 2C and 2D) and can be achieved through coordination of existing targeted sampling efforts or the addition of a few, targeted sampling efforts. This approach (see “Inferences about population parameters” in S1 Text) can be viewed as a variant of “capture–recapture” modeling (“Capture–recapture inference” in S1 Text) and has a limited history of use in epidemiology.

As an example, consider the estimation of prevalence. The primary data source for COVID-19 in many locations is testing of self-reported symptomatic individuals. These data permit direct estimation of the probability that a symptomatic individual is infected. However, tests of asymptomatic individuals will typically be too few and nonrepresentative for useful inference. A targeted random sample can be conducted to estimate the proportion of individuals in the focal population that belongs to each of these 2 groups, symptomatic and asymptomatic individuals. Note that this step requires no additional testing, only a survey of externally detectable symptoms (e.g., temperature readings). The individuals presenting as asymptomatic (or a random subset of them) in this sample can then be tested to estimate infection probability for this group. Estimates of these 3 parameters can be used to estimate prevalence as a derived parameter, or all 3 data sets can be combined within a joint likelihood to estimate the prevalence parameter directly (“Inferences about population parameters” in S1 Text).

Even after dealing with nonrepresentative sampling, counts of individual test results are still influenced by the diagnostic uncertainties of false-positive and negative results. The kind of thinking that underlies the conditional probabilities of infection for individual tests can be incorporated into models for estimating population-level parameters. Replicate testing can be used to deal with diagnostic errors in 2 approaches developed for studying animal populations: occupancy modeling [17] and multi-event capture–recapture modeling [21]. These approaches permit estimation of prevalence, for example, in the face of classification errors. If assessment of symptoms or quantitative measurements of infection status [22] are obtained, they can be incorporated into the modeling as covariates. Elaborations of these modeling frameworks permit error rate parameters to vary temporally or across individuals (see “Dealing with errors in population-level inference” in S1 Text). The occupancy and capture–recapture approaches treat both error rates and focal population parameters as unknown parameters in a single joint likelihood, properly incorporating the various sources of uncertainty in estimates of focal parameters and their variances. Variance estimates of focal parameters are important to decision-making and can be incorporated directly into formal optimization methods designed to deal with such uncertainty.

Prevalence is one parameter of interest, but a central point of this commentary is that sample designs and analysis methods must be tailored to a specified set of focal parameters. Inferences about other key population parameters, such as mortality and infection rates, require repeat testing of the same individuals over time (e.g., as currently done in vaccine trials). Periodic testing is used to assess death or recovery of initially infected individuals and death and infection state for individuals not initially infected. Multi-event capture–recapture models [21] can be used with data on individuals obtained at multiple assessment points, *t*, *t*+1, etc. (e.g., weekly and monthly). At each assessment point, each individual still living from the original sample is tested, and the observed state (e.g., uninfected and infected or susceptible, infected, and recovered) is recorded. The state space can be expanded to include other characteristics of individuals that are relevant to sampling (e.g., symptomatic infected, symptomatic uninfected, asymptomatic infected, and asymptomatic uninfected). The multi-event capture–recapture framework admits state misclassification and provides estimates of the probability of an individual being in a specific state, as well as the state-specific probabilities of death during each interval (e.g., *t* to *t*+1) and making state transitions (e.g., becoming infected or moving to the recovered state).

In the event that all individuals from the initial sample cannot be located to be tested at each assessment point, the modeling approach includes state-specific detection probabilities, recognizing that (1) on some occasions, disease state cannot be assessed for every individual; and (2) state misclassification may occur for individuals that are tested. Detection history data consist of information for each potential assessment or testing period on whether the individual was tested or not, and, if so, what the test outcome was (to what observation state was the individual assigned for that sample period). The data are then modeled as a function of parameters that include detection probabilities, survival probabilities, state transition probabilities, and state classification probabilities. If the initial sample of individuals to be followed is not random or representative, then parameter estimates corresponding to the entire population can be obtained as a weighted sum of estimated probabilities as in expression F in S1 Text. The need to track individuals over time necessitates consideration of patient data protection, as for other COVID-19 processes such as contact tracing. Sampling design and corresponding analytic methods again depend on the objectives of the testing program, which include the focal parameter(s) required to meet population-level objectives.

## Conclusions

Testing thus informs both individual- and population-level control decisions, but different objectives necessitate different sampling strategies—from administering tests to symptomatic individuals appearing at healthcare facilities to preemptively testing and surveying individuals according to a priori designs without regard to presence of symptoms or appearance in the healthcare system. Limited resources require decisions about allocation of tests to inform individual treatment and also public health decision-making. **The keys to successful testing strategies are (1) to clearly specify the objectives of the testing efforts; and (2) to tailor sampling and analytic approaches to those objectives**. Importantly, data produced by testing for one objective may not be useful for other objectives without specific supporting data and associated analytic approaches. Currently, individual-level objectives are prioritized, and testing data are later repurposed to estimate epidemiological parameters and inform public health objectives. In order to accurately estimate population-level parameters, we need to supplement existing testing efforts with small, but coordinated sampling efforts designed with population-level objectives in mind. Data from relatively few tests, when allocated in a coordinated and efficient manner and combined with tailored inference methods, can carry a high value of information, with direct applicability not only to epidemiological model parameterization, but also to decision-making about the pandemic. Clear thinking about test allocation to population-level objectives will be especially important for epidemiological modeling and control of new variants of COVID-19 and for making decisions about vaccine allocation and efficacy globally. Certainly, we are not claiming that such clear thinking does not exist in specific programs and studies being carried out in the US, but rather that we need more of it, especially at a coordinated national level.

Allocation of tests to specific monitoring objectives can be based on current assessments of the relative value of information to the different decisions that the data are intended to inform and the relative importance of these decisions to overall COVID-19 control for individuals and populations. Sampling designs and analyses of resulting data can then be tailored to each objective. Although COVID-19 has brought this issue into stark relief, lack of resources and support for targeted disease monitoring and evaluation programs has been a limitation to the assessment and design of vaccine programs the world over. In much the same way that we should shine new light where we expect our missing keys and not just search where there is available light, we could strengthen current testing approaches in order to better support containment during pandemic vaccine rollout with the strategic allocation of relatively few additional tests and symptom-based surveys.

## Supporting information

S1 Text Supporting manuscript text including (1) A numerical example of misleading testing statistics; (2) Conditional probabilities of infection; (3) Inferences about population parameters; (4) Capture–recapture inference; and (5) Dealing with errors in population-level inference. (DOCX)
