# Supplementary material for: Strategic testing approaches for targeted disease monitoring can be used to inform pandemic decision-making
Source: PLoS Biol. 2021 Jun 17;19(6):e3001307. doi: 10.1371/journal.pbio.3001307 (PMC8241114; doi:10.1371/journal.pbio.3001307)
Supplement: S1 Text — (DOCX) [file pbio.3001307.s001.docx]

**S1 Text**

Supporting Information for

Strategic testing approaches for targeted disease monitoring can be used to inform pandemic decision-making

James D Nichols, Tiffany L Bogich, Emily Howerton, Ottar N Bjørnstad, Rebecca K Borchering, Matthew Ferrari, Murali Haran, Christopher Jewell, Kim M Pepin, William JM Probert, Juliet RC Pulliam, Michael C Runge, Michael Tildesley, Cécile Viboud, Katriona Shea

Correspondence to: [jamesdnichols2@gmail.com](mailto:jamesdnichols2@gmail.com); [tlb927@psu.edu](mailto:tlb927@psu.edu)

Supporting Text

Numerical example of misleading testing statistics

Assume interest in the pathogen prevalence (proportion of individuals in the population that is infected during a relatively short time interval: e.g., 1 week) within 2 different regions for the purpose of comparison. Further assume that both regions have identical proportions of symptomatic and asymptomatic individuals in the population, identical proportions infected in each group, and thus identical overall prevalence. The 2 regions have different testing approaches with region B testing a larger proportion of symptomatic individuals than region A, and even some asymptomatic individuals, whereas region A does no testing of asymptomatic individuals. We compute statistics for perfect tests (no errors in testing) and imperfect tests that admit false negatives and false positives (Table A). For this example, we consider the simplified case of binary rather than continuous quantitative results, but the same concept applies to both cases.

One comparison of interest is that of estimated prevalence versus true prevalence. True prevalence for region A is > 3 times larger than estimated prevalence for tests with and without errors (Table A). Estimated prevalence for region B is somewhat higher than that for region A because of the additional testing, but true prevalence for B is still > 2 times larger than the region B estimates. If we focus on comparing estimated prevalence for the 2 regions, we see that estimated prevalence for region B is over 50% larger than that for region A with both imperfect and perfect testing. It is clear that the rate of testing and the kinds of errors admitted by tests can influence the kinds of summary statistics that are commonly computed to summarize disease dynamics. Epidemiologists recognize the limitations of such statistics, yet such statistics are still presented in public media (such as newspapers and websites), rather than approximately unbiased estimates of focal parameters such as true prevalence.

Conditional probabilities of infection

Approaches to dealing with inferences about infection at the level of the individual require information about the probabilities of false negatives and positives. Define *p_lm_* as the probability that a test result (*x_k_* = *l*) indicates individual *k* to be in disease state *l*, given that true disease state is *m.* If we define state *z_k_* = 1 to mean that individual *k* is infected and state *z_k_* = 0 to mean uninfected, then *p*_11_ is the probability of correctly detecting infection when present, and its complement (1- *p*_11_) is the probability of a false negative. Similarly, *p*_10_ is the probability of a false positive, incorrectly declaring an individual in state *z_k_* = 0 to be infected. Because these probabilities are conditional on the unknown true state of the tested individual, statements about the likelihood of true infection are also conditional on the underlying pathogen prevalence (i.e., probability that a randomly selected individual is infected, *ψ*).

The conditional probability that an individual testing positive is actually infected (termed “positive predictive value” by [23]) can be written as:

$\Pr\left( z_{k}=1 | x_{k}=1 \right)=\frac{\psi p_{11}}{\psi p_{11}+(1-\psi)p_{10}}$. (A)

The numerator of (A) is the probability that an individual is infected and tests positive for the disease, whereas the denominator is the sum of the numerator probability and the probability that the individual is not infected, but the test results in a false positive. The complement of (A) is the probability of an error: the individual is not infected but tests positive.

Examples computed using (A) should correspond well to intuition. Consider a situation in which *p*_11_ = 0.9 and *p*_10_ = 0.02. If the disease prevalence in a sampled location is moderate, e.g., *ψ* = 0.01, then the probability that a positive test result is an error is relatively high, about 0.69. But if disease prevalence is much higher, say *ψ* = 0.10, then the probability of an incorrect positive test result is much smaller at 0.17. The conditional probability that an individual testing negative is truly not infected (termed “negative predictive value” by [23]) can be written similarly as:

$\Pr\left( z_{k}=0 | x_{k}=0 \right)=\frac{(1-\psi)(1-p_{10})}{\psi{(1-p}_{11})+(1-\psi){(1-p}_{10})}$ . (B)

The complement of (B) is the probability that the negative test result is false.

Note that some types of tests (e.g., some serological tests) may yield false negatives but do not admit false positives. In this case, we set *p*_10_ in the above expressions to 0. Expression (A) reduces to 1; that is in the absence of false positives, a positive test result means that an infection is present with no uncertainty. Expression (B) reduces to:

$\Pr\left( z_{k}=0 | x_{k}=0 \right)=\frac{(1-\psi)}{\psi{(1-p}_{11})+(1-\psi)}$ . (C)

Use of (A) and (B) to compute probabilities of infection, given either a positive or negative test, should be useful when considering any of the reasons for seeking individual-level inferences; treatment of the focal individual, safety of the healthcare workers attending the focal individual, or contact tracing. If the computed probabilities admit more uncertainty than deemed acceptable, then they can be supplemented with additional information provided by replicating tests (e.g., see [24]). Replicate tests would be conducted at the same time as the original test (e.g., use of two swabs and associated laboratory analyses, instead of just one). Consider the complement of (C), representing the probability of no positive test result, given that the true state is infected:

$\Pr\left( z_{k}=1 | x_{k}=0 \right)=\frac{\psi{(1-p}_{11})}{\psi{(1-p}_{11)}+(1-\psi)}$ . (D)

If *p*_11_ = 0.8 and *ψ* = 0.1, then the probability that the individual is infected, given that a single test was negative, is 0.02. Expression (D) can be written generally for *g* tests as:

$\Pr\left( z_{k}=1 | \boldsymbol{x}_{\boldsymbol{k}}=0, 0\ldots0 \right)=\frac{\psi{(1-p_{11})}^{g}}{\psi{(1-p_{11)}}^{g}+(1-\psi)}$ , (E)

where ***x_k_*** = 0, 0, …0 is a vector of *g* 0’s, and $\left( 1-p_{11} \right)^{g}$ is the probability of failing to detect the infection in all *g* tests. If *g* = 2 tests are conducted on an individual and both are negative, then the probability that the individual is infected is only 0.004. So replicated testing can provide a means of reducing diagnostic uncertainty. Expression (E) is based on the assumption of independence of the multiple tests administered to the same individual, but this assumption can be relaxed in various ways (see below).

In some cases, developers of testing programs may face the choice of selecting inexpensive tests with large error rates or more expensive tests with lower error rates. The above expressions for computing conditional probabilities of infection can be used to compute accuracy associated with a specific cost. For example, if one test is twice as expensive as another, then we can simply compute the conditional infection probabilities given no infection detected (E) and compare the result for a single test of the more expensive test (larger *p*_11_) with that for 2 tests of the less expensive test (smaller *p*_11_). Because this conditional probability represents an error (false negative), the test with the smaller conditional probability is preferable.

The above expressions are based on single probabilities of making false negative (1-*p*_11_) and false positive (*p*_10_) errors. However, in the case of false negative errors, *p*_11_ might vary across individuals for reasons such as current viral load, time since infection, etc. In situations where such variation is associated with individual covariates (e.g., symptomatic individuals and asymptomatic individuals might have different values for *p*_11_), the logit of the detection parameter (*p*_11_) can be modeled as a function of the covariates. In the case of heterogeneity not associated with a measurable covariate, *p*_11_ can be modeled as a finite [25] or continuous [26] mixture. These mixture approaches are similar to the frailty concept in epidemiological competing risk modeling of mortality rates (e.g., [27-28]). Heterogeneous error probabilities represent one of multiple ways in which we might want to relax assumptions underlying expressions (A) through (E) (for this and other approaches see [17, 29-31]).

Heterogeneous error probabilities may cause a lack of independence for multiple tests of the same individual, in the sense that all tests for one individual may have error probabilities that are lower than average and all for another individual are higher than average. If individual covariates are associated with this variation, then they can be incorporated into models for estimation of single-test false positive or negative error probabilities and used with equation (E) to estimate individual-specific probabilities of making a specific error in multiple tests. In the absence of covariates, the mixture models for heterogeneous errors can be used to estimate multi-test error probabilities that apply to specified portions of the population (e.g., applicable to 90% of individuals). Finally, if non-independence of tests is instead associated with the outcomes of previous tests, the so-called “removal” models (see [17, 29]) provide one approach to inference, providing estimates of one error probability for tests administered before the first “detection” and a different error probability for tests administered after initial detection.

We note that the dependence of these conditional probabilities (Expressions A through E) on prevalence, *ψ*, requires reasonably accurate estimates of this parameter. The methods outlined in “Capture-recapture inference” in S1 Text can be used to estimate prevalence, even in cases where both false positives and false negatives are possible. In contrast, such errors can lead to substantial biases in estimates of prevalence that assume no errors, producing poor estimates of these conditional probabilities, and thus poor evidence for making decisions. In the above expressions, the underlying probability that a randomly selected individual is infected is treated as a constant, *ψ.* However, if known individual covariates associated with pathogen presence can be identified, then they can be used with data from multiple individuals to estimate individual-specific probabilities of infection, *ψ_k_*. For example, presence or absence of key COVID-19 symptoms could be used to model *ψ_k_* (e.g., using a linear-logistic approach with occupancy modeling, [17]) and produce more precise estimates of the conditional probabilities for tested individuals.

All of the above expressions, A through E, are presented as functions of probabilities of pathogen prevalence, *ψ*, and detection-classification, *p_lm_*, and can be computed using estimates of these parameters. However, it will often be more efficient to analyze the data from a particular group of tests (e.g., from a single location in a single day or week) together using an occupancy modeling framework [17]. As long as some individuals receive replicate tests (this information is needed to estimate error rates), this approach permits direct estimation of all prevalence and detection-classification parameters, the above derived conditional probabilities, and all associated variances, for example using program PRESENCE [17]. If no replicate testing is available, then information about detection-classification parameters must come from special trials or laboratory studies, and the data from these studies can be incorporated into the likelihood in order to again compute all parameter estimates in a single analysis. Individual, time-specific and location-specific covariates hypothesized to influence prevalence parameters or detection-classification parameters, or both, can be included as ancillary data in these analyses and their importance assessed via model selection or likelihood ratio tests. The modeling framework and user-friendly software for these kinds of analyses are available [17].

Inferences about population parameters

*Prevalence.* This term is defined as either the number or fraction of individuals infected in a population at a given point in time. Here we focus on the latter definition and attempt to estimate the probability that a randomly selected individual from an exposed population is infected (denote as *ψ*). This latter probability can be estimated by selecting a random or representative (defined with respect to factors influencing the likelihood that an individual is infected, e.g., age, sex, prior health condition, etc.) sample of individuals and testing them. The fraction of individuals from such a sample that tests positive for the disease is an estimate of *ψ* (denote as $\hat{\psi}$, where the hat denotes an estimate), with variance *ψ*(1-*ψ*)/*n*, where *n* is the number of individuals in the tested sample). If *N* is the known number of individuals in the focal population, then the total number of infected individuals in the population (denote as $N^{i}$) can be estimated as $\hat{N}^{i}=\hat{\psi}N.$ If total number of individuals in a population is not known, but estimated, then $\hat{N}^{i}=\hat{\psi}\hat{N}$.

When it is not possible to obtain large samples of individuals in a random or representative manner, an alternative approach is to select groups of individuals in a nonrepresentative way and to separately estimate the probabilities that a randomly sampled individual would appear in these groups. One class of methods developed to implement this approach is often labeled as “capture-recapture” modeling (e.g., [29, 32]). These models were developed in the wildlife sciences and have a long, but still limited, history of use in epidemiology [33-37]. Because COVID-19 testing to-date has focused primarily on symptomatic individuals self-reporting to the healthcare system, thus producing large samples, we could estimate the probability that a symptomatic individual is infected as the fraction of symptomatic individuals that tests positive (denote as $\hat{\pi}^{s})$. We could then select a random or representative sample of asymptomatic individuals to test and estimate the probability that an asymptomatic is infected (denote as $\hat{\pi}^{a})$. Finally, we could estimate the probability that a randomly selected individual is symptomatic using a potentially much larger sample (as this sample requires no testing) by simply assessing symptoms. The fraction of symptomatic individuals in this sample then estimates $\theta$, the probability that a randomly selected individual is symptomatic. The probability that a randomly selected individual in the population is infected could then be estimated as:

$\hat{\psi}=\hat{\theta} \hat{\pi^{s}}+\left( 1-\hat{\theta} \right)\hat{\pi^{a}}$ . (F)

The above expression (F) assumes the same probability of testing positive for all symptomatic individuals. However, it is possible for example, that variation in access to healthcare may result in symptomatic individuals with good access having different (likely lower) probabilities of being infected than individuals with poor access, who must expend greater effort to travel to locations in order to be tested. The samples used to estimate the probability of infection for symptomatic individuals can be used to estimate probability of testing positive as a function of distance to nearest testing facility, for example. If such variation is found to exist, then more complicated versions of (F) would be required that incorporate this source of variation.

A specific problem that is relevant to the general issue of nonrandom and non-representative sampling is refusal of a selected person to take a SARS-CoV-2 test. Denote as *π^v^*, the fraction of randomly selected individuals that refuses to take a test. If the individuals in this group are similar to the (1- *π^v^*) individuals with respect to factors influencing the parameter of interest, then this presents no problem, and inference is simply based on those tested. If persons selected for testing that do and do not choose to take a test differ with respect to known factors influencing the presence of the pathogen, then covariate relationships for those factors estimated from tested individuals can be used to estimate pathogen presence in the individuals who refuse testing.

In the case of possible differences in disease prevalence between persons that do and do not submit to testing, that are not known to be associated with measurable traits, it may be possible to reduce nonresponse to negligible levels via rewards. For example, consider a testing program in which every 50^th^ person selected is given a reward conditional on participation. Many of these individuals would have participated even without the reward, whereas a subset would have refused had there been no reward offered. The prevalence for the 2 samples (those offered a reward and those not) can be compared. If found to be different, then the population prevalence is best estimated using the reward sample only, whereas if the prevalence values are similar, then the combined sample can be used.

*Infection rate.* We define a finite (contrasted with instantaneous) infection rate at time *t* in location *j* as the probability that a randomly selected individual who is not infected at time *t* is infected at time *t* +1, where the time step (e.g., perhaps 1 day or 1 week) is selected relative to the known or hypothesized speed of infection. A random selection of individuals at location *j* is tested at time *t*, yielding $n_{j,t}^{u}$ individuals who do not test positive. These individuals are retested at time *t*+1, yielding $n_{j,t+1}^{i}$individuals that are newly infected. The infection rate can then be estimated as the newly infected at *t*+1 divided by the number not infected at *t*, $n_{j,t+1}^{i}/n_{j,t}^{u}$. Monitoring for the purpose of developing risk maps may focus on the relationship between infection rate and such factors as local prevalence. A likelihood could be developed for the data, and infection rate modeled as a function of prevalence probability. The resulting relationship can be used to predict infection rates at locations for which such periodic retesting is not possible. If estimation of this relationship is a primary focus of monitoring, an effort should be made to select testing locations predicted to show substantial variation in prevalence.

*Mortality/case fatality rate.* Disease-specific mortality rate can be defined as the probability that an individual infected with a pathogen dies as a result of the infection. One approach to inference would be to first select a random or representative sample of individuals to be tested. The confirmed infected individuals within this sample (denote as $n^{i}$) are then followed until death or recovery. Mortality rate (*m*) can then be estimated as the ratio of number of deaths (*d*) to number infected, $\hat{m}=d/n^{i}$. While individuals from any sampled location should be selected randomly or representatively for testing, the location itself need not be selected randomly. As a practical matter, it is usually wise to select the initial random sample of people from a location believed to be a hotspot or area of high prevalence, as the variance of $\hat{m}$ is $\frac{m\left( 1-m \right)}{n^{i}}$, becomes smaller with larger sample size of infected individuals.

Capture-recapture inference

Capture-recapture sampling was developed in the disciplines of wildlife biology and animal ecology to deal with the reality that virtually no sampling method for wild animals (e.g., direct observation, capture) yields total counts of animals in the sampled location, instead providing counts representing unknown fractions of animals present (counts always “miss” animals). The capture-recapture approach to dealing with this issue is to try to estimate the detection probability, that is the probability that an animal present at a location will appear in the count, and then use this probability and the count to draw inference about the true number of animals present (e.g., [29, 32]).

The first such estimator was developed by LaPlace [38] to estimate the human population of France, and was later developed independently by Lincoln [39] to estimate the waterfowl population of North America. Lincoln [39] was charged with developing hunting regulations for North American ducks, so focused on the population size in late summer and early fall, just before the hunting season. A harvest survey monitoring program in the U.S. provided an estimate of total number of ducks killed or harvested, *H.* So Lincoln [39] estimated the probability that a bird alive just before the hunting season appeared in that sample of harvested birds from duck banding data. Specifically, ducks were banded in late summer and early fall across the North American breeding grounds and many were shot by hunters and reported to the U.S. Bureau of Biological Survey. The fraction shot and reported, *h*, was viewed as a sampling probability and used to estimate total population size in late-summer – early fall as:

$\hat{N}= \frac{\hat{H}}{\hat{h}}$, (G)

where the hats denote estimates.

One use of capture-recapture thinking for COVID-19 monitoring will be to deal with nonrandom or non-representative sampling. Lincoln’s [39] estimator was later used to estimate the age ratio (young to adult; an index of reproductive rate) in the late-summer early-fall duck population. A naïve approach to this inference would have been to compute the ratio of young to adult in the harvested birds comprising *H*. However, it was well known that young ducks have a greater probability of being shot than adults (*h^y^* >*h^a^*), where the superscript denotes age; *y =* young, *a* = adult. Use of (G) with data from each age class separately provides estimates of age-specific abundance that can then be used to estimate the age ratio, *A,* as:

$\hat{A}=\frac{\hat{N^{y}}}{\hat{N^{a}}}=\frac{{\hat{H^{y}}}/{\hat{H^{a}}}}{{\hat{h^{y}}}/{\hat{h^{a}}}}$ . (H)

This estimator (H) thus deals with the different sampling probabilities associated with young and adults by estimating them directly from the banding data, permitting unbiased estimation of the focal parameter, age ratio [29].

This basic approach to inference in the face of non-representative sampling is now widely used in other disciplines (e.g., [36, 40]) and can be readily used in COVID-19 monitoring. As one of many possible scenarios, assume that we are monitoring number of cases among asymptomatic individuals over some timeframe in a local community and want to estimate the total number of COVID-19 cases for asymptomatic individuals for that time/place. Let *n^ai^* be the count of asymptomatic individuals tested and found to be infected in the community over the specified timeframe. Using the approach and notation for expression (F), we could select a random or representative sample of asymptomatic individuals to test and directly estimate the probability that an asymptomatic individual is infected (denote as $\hat{\pi}^{a}$; to simplify, assume error-free tests). We could estimate the probability that an individual is symptomatic using a potentially larger sample by simply assessing symptoms of individuals selected randomly. The fraction of asymptomatic individuals in this sample then estimates 1-$\theta$, the probability that a randomly selected individual is not symptomatic. The estimated number of asymptomatic cases in the community is then estimated as:

$\hat{N^{ai}}= \frac{n^{ai}}{(1-\hat{\theta} )\hat{\pi}^{a}}$ .

The denominator of this estimator is simply the estimated probability that an asymptomatic individual from the population is selected for testing *and* is infected. If testing is imperfect (i.e., false negatives are possible), then the denominator of this expression must include this error probability as well.

Dealing with errors in population-level inference

*Prevalence.* Inferences about the probability that a randomly selected individual from an exposed population is infected (*ψ*) are often based on random or representative samples of individuals who are then tested. In the presence of misclassification (false negatives, false positives), the fraction of individuals from such a sample testing positive for the disease does not necessarily provide a good estimate of *ψ.* For example, in the case of only false negative errors (1-$p_{11}$), the expected value for fraction of individuals testing positive will be $\psi p_{11}.$

Two well-developed and closely related approaches for dealing with both false positives and negatives are occupancy modeling [17, 41] and multi-event capture recapture modeling [21]. Both approaches use replication to derive the extra information required to deal with errors, where replicate sampling usually occurs within a very short time period (e.g., same day as original test or at least within 1-2 days), such that infection state is not expected to change between sampling “occasions”. For example, under an occupancy framework, we might test each individual in the sample twice (*g* = 2). If “1” denotes a positive test and “0” a negative test result, then each sampled individual (*k*) would exhibit one of the following detection histories (*x_k_*): 11, 10, 01, 00; where the first and second numbers of each pair denote the results of the first and second tests, respectively. The likelihood required for inference is based on the probabilities associated with each history. When tests do not admit false positives, we can write the probability for history (10), for example, as

$\Pr\left( x_{k}=(10) | \psi,p_{11} \right)=\psi p_{11}\left( 1-p_{11} \right)$ (I)

In the absence of false positives, the positive result of the first test tells us that the individual is infected, *ψ*, and infection was then detected at the first test and not the second, *p*_11_(1-*p*_11_).

In the case of no positive test results, there are two possibilities that must be modeled,

$\Pr\left( x_{k}=(00) | \psi,p_{11} \right)=\psi{(1-p_{11})}^{2}+(1-\psi).$ (J)

The first term is the probability that the individual is infected but experienced two successive false negative errors. The second term is the probability that the individual was not infected. The likelihood for a sample of replicate tests for *n* individuals is then:

$L(\psi,p_{11}|{\{x}_{k}\})=\prod_{k=1}^{n} Pr(x_{k})$ . (K)

In this situation, where only false negatives are possible, a more efficient sampling scheme is to not proceed to an additional replicate test after an initial positive result is found. This kind of sampling changes the likelihood slightly but permits unbiased inference (see removal design of [17]).

The above expressions are based on single probabilities of making false negative (1-*p*_11_) and false positive (*p*_10_) errors. As described in “Conditional probabilities of infection” in S1 Text, in the case of false negative errors, *p*_11_ might vary across individuals for reasons such as current viral load, time since infection, etc. As described in “Conditional probabilities of infection” in S1 Text, such heterogeneity can be dealt with via covariates or mixture models (e.g., [25, 26]). When individuals are followed over time for periodic (e.g., weekly) testing of disease state dynamics ( “Inferences about population parameters” in S1 Text), replicate testing at each sampling point would permit inferences about trends in *p*_11_ as a function of time since infection. The main point is that there are many extensions of the framework presented above designed to deal with error rate parameters that vary across individuals and/or time.

In the more general case of false positive and false negative errors, the modeling includes the extra probability, $p_{10}$, of making a false positive error. Models for this situation were first developed by Royle and Link [42]. Strongest inferences are possible when there is a subset of individuals for which true infection state is known (e.g., [17, 43]). Various sampling designs permit inferences in the presence of both false positives and negatives [44], with the “calibration design” most likely to be useful for disease surveillance. Under this design, most of the information about classification probabilities, *p*_11_ and *p*_10_, is obtained with laboratory tests of samples of known infection state. In this case, the replication occurs within the laboratory testing and not with the field testing of individuals.

Estimates of the probability of being infected, $\hat{\psi}$, obtained using occupancy modeling are applicable to the samples of individuals that are tested. If individuals are sampled randomly or representatively from a population of interest, then the estimated prevalence should also pertain to the focal population. However, if estimates are based on a nonrepresentative sample (e.g., large number of symptomatic individuals and a much smaller number of asymptomatic individuals), then capture-recapture or other methods are required to deal with the different probabilities of symptomatic individuals and asymptomatic individuals appearing in the sample of tested individuals. One approach would be to estimate the probability of being infected separately for symptomatic individuals and asymptomatic individuals and to then obtain an overall weighted average estimate, $\hat{\psi}$, where the weights are the estimated fractions of symptomatic individuals and asymptomatic individuals in the focal population (see expression F). The important point is that proper inference requires attention to both sampling design and infection state classification.

*Infection rate and disease-induced mortality rate.* Inferences about both mortality and infection rates begin with a sample of tested individuals who are then followed over time in order to assess death or recovery of initially infected individuals, and death (presumed unlikely with respect to the study time scale) and infection state for individuals not initially infected. Multi-event capture-recapture models [21] can be used with resulting data that are organized as individual testing histories. At multiple assessment points (e.g., weekly, monthly), *t*, *t*+1, etc., each individual still living is tested, and the observed state (e.g., uninfected, infected; or susceptible, infected, recovered) is recorded. For example, under an SIR (susceptible, infected, recovered) classification, an individual testing history might be as given in the table below indicating that the individual was classified as susceptible for assessment points 1 and 2, not tested at assessment point 3, classified as infected for points 4 and 5 and then recovered for point 6.

| Assessment time point | 1 | 2 | 3 | 4 | 5 | 6 |
| --- | --- | --- | --- | --- | --- | --- |
| Testing result | S | S | – | I | I | R |

In cases where estimated parameters are to be used to inform other data sources, the state space can be expanded to include other characteristics of individuals that are relevant to sampling (e.g., symptomatic infected, symptomatic uninfected, asymptomatic infected, asymptomatic uninfected). Sampling or focal parameters can also be modeled as functions of individual-specific or location-specific covariates. The framework admits state misclassification and provides estimates of the probability of an individual being in a specific state (e.g.,$\hat{\psi_{t}}$ when infected and uninfected are the only states), as well as the state-specific probabilities of dying during the interval *t* to *t*+1 and making state transitions (e.g., becoming infected, S to I transition, or moving to the recovered state, I to R transition). In the event that an individual from the initial sample cannot be located to be tested at each assessment point (indicated by the “-“ in the above testing history), the modeling approach includes state-specific detection probabilities, recognizing that on some occasions disease state cannot be assessed for every individual. State misclassification is incorporated directly into the estimation as well. Parameters used in the modeling include detection probabilities, survival probabilities, state transition probabilities and state classification probabilities. If the initial sample of individuals to be followed is not random or representative, then population-level parameter estimates can be obtained as weighted sums of estimated probabilities as in expression (F).

References (complete reference list, including main text references 1-22)

1. Koff WC, Schenkelberg T, Williams T, Baric RS, McDermott A, Cameron CM, Cameron MJ, Friemann MB, Neumann G, Kawaoka Y, Kelvin AA. Development and deployment of COVID-19 vaccines for those most vulnerable. Science Translational Medicine. 2021 Feb 3;13(579).
2. Kupferschmidt, K., & Vogel, G. Vaccine link to serious clotting disorder firms up. Science, 2021 Apr 16; 372(6539), 220–221. <https://doi.org/10.1126/science.372.6539.220>
3. Emanuel EJ, Persad G, Upshur R, Thome B, Parker M, Glickman A, Zhang C, Boyle C, Smith M, and Phillips JP. Fair allocation of scarce medical resources in the time of COVID-19. N Engl J Med 2020 May 21; 382: 2049-2055.
4. Tegally H, Wilkinson E, Lessells RJ, Giandhari J, Pillay S, Msomi N, Mlisana K, Bhiman JN, von Gottberg A, Walaza S, Fonseca V. Sixteen novel lineages of SARS-CoV-2 in South Africa. Nature Medicine. 2021 Mar;27(3):440-6.
5. White House. National Security Memorandum on United States Global Leadership to Strengthen the International COVID-19 Response and to Advance Global Health Security and Biological Preparedness. 2021 Jan 21. <https://www.whitehouse.gov/briefing-room/statements-releases/2021/01/21/national-security-directive-united-states-global-leadership-to-strengthen-the-international-covid-19-response-and-to-advance-global-health-security-and-biological-preparedness/>
6. Riley S, Ainslie KE, Eales O, Walters CE, Wang H, Atchison C, Fronterre C, Diggle PJ, Ashby D, Donnelly CA, Cooke G. Resurgence of SARS-CoV-2: detection by community viral surveillance. Science. 2021 Apr 23;372(6545):990-995
7. Tromberg BJ, Schwetz TA, Pérez-Stable EJ, Hodes RJ, Woychik RP, Bright RA, Fleurence RL, Collins FS. Rapid scaling up of Covid-19 diagnostic testing in the United States—the NIH RADx initiative. New England Journal of Medicine. 2020 Sep 10;383(11):1071-7.
8. Botti-Lodovico Y, Rosenberg E, Sabeti PC. Testing in a Pandemic—Improving Access, Coordination, and Prioritization. New England Journal of Medicine. 2021 Jan 21;384(3):197-9.
9. Mina MJ, Parker R, Larremore DB. Rethinking Covid-19 test sensitivity—A strategy for containment. New England Journal of Medicine. 2020 Nov 26;383(22):e120.
10. Paltiel AD, Zheng A, Walensky RP. Assessment of SARS-CoV-2 screening strategies to permit the safe reopening of college campuses in the United States. JAMA network open. 2020 Jul 1;3(7):e2016818.
11. Yoccoz NG, Nichols JD, Boulinier T. Monitoring of biological diversity in space and time. Trends in ecology & evolution. 2001 Aug 1;16(8):446-53.
12. Nichols JD, Williams BK. Monitoring for conservation. Trends in ecology & evolution. 2006 Dec 1;21(12):668-73.
13. Pacifici K, Dorazio RM, Conroy MJ. A two‐phase sampling design for increasing detections of rare species in occupancy surveys. Methods in Ecology and Evolution. 2012 Aug;3(4):721-30.
14. Chipeta MG, Terlouw DJ, Phiri KS, Diggle PJ. Adaptive geostatistical design and analysis for prevalence surveys. Spatial Statistics. 2016 Feb 1;15:70-84.
15. Probert WJ, Shea K, Fonnesbeck CJ, Runge MC, Carpenter TE, Dürr S, Garner MG, Harvey N, Stevenson MA, Webb CT, Werkman M. Decision-making for foot-and-mouth disease control: objectives matter. Epidemics. 2016 Jun 1;15:10-9.
16. Watson J, Whiting PF, Brush JE. Interpreting a covid-19 test result. Bmj. 2020 May 12;369.
17. MacKenzie, D.I., J.D. Nichols, J.A. Royle, K.H. Pollock, L.A. Bailey, and J.E. Hines. Occupancy modeling and estimation. 2nd edition. San Diego, CA: Academic Press; 2018.  641pp.
18. Skittrall JP, Fortune MD, Jalal H, Zhang H, Enoch DA, Brown NM, Swift A. Diagnostic tool or screening programme? Asymptomatic testing for SARS-CoV-2 needs clear goals and protocols. The Lancet Regional Health-Europe. 2021 Feb 1;1.
19. Wu SL, Mertens AN, Crider YS, Nguyen A, Pokpongkiat NN, Djajadi S, Seth A, Hsiang MS, Colford JM, Reingold A, Arnold BF. Substantial underestimation of SARS-CoV-2 infection in the United States. Nature communications. 2020 Sep 9;11(1):1-0.
20. Flaxman S, Mishra S, Gandy A, Unwin HJ, Mellan TA, Coupland H, Whittaker C, Zhu H, Berah T, Eaton JW, Monod M. Estimating the effects of non-pharmaceutical interventions on COVID-19 in Europe. Nature. 2020 Aug;584(7820):257-61.
21. Pradel R. Multievent: an extension of multistate capture–recapture models to uncertain states. Biometrics. 2005 Jun;61(2):442-7.
22. Hay JA, Kennedy-Shaffer L, Kanjilal S, Lipsitch M, Mina MJ. Estimating epidemiologic dynamics from single cross-sectional viral load distributions. MedRxiv. 2020 Jan 1.
23. CDC Interim Guidelines for COVID-19 Antibody Testing in Clinical and Public Health Settings 2020. Updated 2020 August. 2020.
24. Larremore, D.B., Wilder, B., Lester, E., Shehata, S., Burke, J.M., Hay, J.A., Tambe, M., Mina, M.J. and Parker, R. Test sensitivity is secondary to frequency and turnaround time for COVID-19 surveillance. MedRxiv. 2020.
25. Pledger, S. Unified maximum likelihood estimates for closed capture–recapture models using mixtures. Biometrics. 2000;56(2):434-442.
26. Dorazio, R.M. and Royle, A.J. Mixture models for estimating the size of a closed population when capture rates vary among individuals. Biometrics. 2003;59(2):351-364.
27. Hougaard, P. Life table methods for heterogeneous populations: distributions describing the heterogeneity. Biometrika. 1984;71(1):75-83.
28. Hougaard, P. Survival models for heterogeneous populations derived from stable distributions. Biometrika. 1986;73(2):387-396.
29. Williams, B.K., Nichols, J.D. and Conroy, M.J. Analysis and management of animal populations. Academic Press; 2002.
30. Kery, M. and Royle, J.A. Applied Hierarchical Modeling in Ecology: Analysis of Distribution, Abundance and Species Richness in R and BUGS: Volume 1: Prelude and Static Models. Academic Press; 2016.
31. Kery, M. and Royle, J.A. Applied Hierarchical Modeling in Ecology: Analysis of Distribution, Abundance and Species Richness in R and BUGS: Volume 2: Dynamic and Advanced Models. Academic Press;2020.
32. Seber, G.A.F. The estimation of abundance and related parameters. London: Griffin;1982.
33. Wittes, JT and VW Sidel. A generalization of the simple capture-recapture model with applications to epidemiological research. J Chronic Diseases. 1968;21:287–301.
34. LaPorte, RE, DJ McCarty, ES Tull, and N Tajima. Counting birds, bees, and NCDs. Lancet. 1992;339:494–495.
35. Chao, A, PK Tsay, S-H Lin, W-Y Shau, and D-Y Chao. The applications of capture-recapture models to epidemiological data. Statistics in Medicine. 2001;20: 3123–3157.
36. Nichols, J.D. Confronting uncertainty:  Contributions of the wildlife profession to the broader scientific community. J. Wildl. Manage. 2019;83:519-533.
37. Tabak, M.A., Pedersen, K., Miller, R.S., Detection error influences both temporal seroprevalence predictions and risk factors associations in wildlife disease models. Ecology and Evolution. 2019;9: 10404-10414.
38. LaPlace, P.S. “Mémoire sur la Figure de la Terre”. In Mémoires de l'Académie Royale des Sciences de Paris. 1786;17–46. Reprinted in his Oeuvres, vol.11, Gauthier-Villars et Fils, Paris, 1895: 3-32.
39. Lincoln, F.C. Calculating waterfowl abundance on the basis of banding returns (No. 118). US Department of Agriculture;1930.
40. Horvitz, D.G., and Thompson, D.J. A generalization of sampling without replacement from a finite universe. Journal of the American Statistical Association. 1952;47:663-685.
41. MacKenzie, D.I., Nichols, J.D., Lachman, G.B., Droege, S., Andrew Royle, J. and Langtimm, C.A.. Estimating site occupancy rates when detection probabilities are less than one. Ecology. 2002;83(8): 2248-2255.
42. Royle, J.A. and Link, W.A. Generalized site occupancy models allowing for false positive and false negative errors. Ecology. 2006;87(4): 835-841.
43. Miller, D.A., J.D. Nichols, B.T. McClintock, E.H.C. Grant, L.L. Bailey, and L. Weir. Improving occupancy estimation when two types of observational error occur: nondetection and species misidentification. Ecology. 2011;92:1422-1428.
44. Chambert, T., Miller, D.A. and Nichols, J.D. Modeling false positive detections in species occurrence data under different study designs. Ecology. 2015;96(2): 332-339.

**Table A.** Comparative estimates of prevalence for 2 populations characterized by identical proportions infected, but different testing approaches.

|  | Region A | Region B |
| --- | --- | --- |
| *Population statistic (proportion of entire population)* | | |
| Asymptomatic infected | 0.02 | 0.02 |
| Asymptomatic uninfected | 0.95 | 0.95 |
| Symptomatic infected | 0.02 | 0.02 |
| Symptomatic uninfected | 0.01 | 0.01 |
| Infected (prevalence) | 0.04 | 0.04 |
| Testing rate (proportion of group tested) | | |
| Asymptomatic | 0.00 | 0.02 |
| Symptomatic | 0.60 | 0.90 |
| Estimated prevalence*^a^* | | |
| Perfect test (*p*_11_ = 1.0, *p*_10_ = 0.0)*^b^* | 0.0120 | 0.0184 |
| Prevalence B / Prevalence A | 1.53 | |
| Imperfect test (*p*_11_ = 0.9, *p*_10_ = 0.02) | 0.0109 | 0.0171 |
| Prevalence B / Prevalence A | 1.57 | |

*^a^* Estimated as total confirmed cases divided by population size. An alternative estimator (also inaccurate in this example) is computed as the fraction of confirmed cases among all individuals tested.

*^b^* *p*_11_ = probability of a positive test result for an infected individual (complement of false negative); *p*_10_ = probability of a positive test result for an uninfected individual (false positive).
